# Outcome of Acute Appendicitis During COVID-19 Infection

**DOI:** 10.7759/cureus.72052

**Published:** 2024-10-21

**Authors:** Ahmed Elmoraly, Jasreen Kaur Sandhu, Jennifer A Thomas, Kapilraj Ravendran

**Affiliations:** 1 General Medicine, East Sussex Healthcare NHS Trust, Hastings, GBR; 2 General Surgery, The Dudley Group NHS Foundation Trust, London, GBR; 3 Nephrology, Government Medical College, Amritsar, Amritsar, IND; 4 Emergency Department, University Hospital of North Midlands, North Midlands, GBR; 5 Rheumatology, Royal National Orthopaedic Hospital, London, GBR

**Keywords:** appendicitis, covid-19, outcome of appendicectomy, outcomes, surgery

## Abstract

Acute appendicitis is the main cause of urgent abdominal surgeries performed worldwide. The disease is most prevalent in the second and third decades of life, and patients typically range in age from five to 45. One of the most common non-elective procedures performed by general surgeons, appendectomy is still the gold standard for treating acute appendicitis despite the variety of clinical presentation patterns. Our study aimed to compare the outcomes of surgically treating acute appendicitis (open versus laparoscopic) with respect to pain control, hospital stay, sepsis, and clinical improvement during the COVID-19 pandemic. This retrospective cohort study audit was conducted at the Dudley Group NHS Foundation Trust. The documents maintained in the form of discharge notes, drug charts, operation notes, admission notes, and sunrise system notes. The Dudley Group NHS Foundation Trust's medical ethics committee approved the study and determined that informed consent was not necessary. The patients' identities remained secret. Data from November 2019 to July 2021 was collected retroactively and analyzed using Excel (Microsoft, Redmond, WA, USA). The gender ratio of patients who had laparoscopic appendectomy was nearly equal, with 21 (52%) and 19 (48%) of the patients who underwent open appendectomy being female (65% of open cases) and 14 (35%) of the cases male (open cases). The laparoscopic group had a higher overall postoperative complication rate (22% versus 18%), but the open appendectomy group had a higher rate of open wound infection. Finally, our study highlights the importance of a nuanced understanding of patient demographics and clinical presentations when comparing open and laparoscopic appendectomy, providing insightful information about the outcomes of acute appendicitis. The differences in postoperative care and complication rates highlight how difficult surgical approach decision-making can be. More investigation is necessary to determine the fundamental causes of these variations and to improve recommendations for the best possible patient care, particularly in the face of difficult medical situations like the COVID-19 pandemic.

## Introduction

The primary reason for urgent abdominal surgeries around the world is acute appendicitis [1,2]. Patients usually fall within the age range of five to 45, with the disease being most common in the second and third decades of life [3]. Even with the variety of clinical presentation patterns, appendectomy remains the gold standard for treating acute appendicitis and is one of the most common non-elective procedures that general surgeons perform [3,4].

In terms of the surgical method, the open traditional laparotomic appendectomy, also known as the "McBurney incision," was initially described by Charles McBurney in 1889 and was for more than a century regarded as the most effective way to treat acute appendicitis [4,5]. The procedure involves making an incision in the lower right quadrant of the abdomen, exposing the colon and part of the appendix [4,6]. Kurt Semm, a German gynecologist, was the first to introduce the minimally invasive laparoscopic appendectomy procedure in 1981 [4,7].

The benefits of laparoscopy for appendicitis include shorter hospital stays, an earlier return to social and occupational activities, a decreased risk of wound infections, a need for fewer postoperative painkillers, a quicker recovery period overall, and improved cosmetic outcomes [4]. However, the results of multiple retrospective studies, meta-analyses, and randomized trials have been inconsistent [4].

Before the pandemic, the general consensus was that, when treating acute appendicitis, laparoscopic appendicectomy was the better surgical option than an open procedure [8]. The UK Joint Royal Colleges released guidelines for surgeons operating during the COVID-19 pandemic on March 26, 2020 [9]. The guidelines advise exercising extreme caution when performing any laparoscopic surgery due to the possibility of aerosol formation during pneumoperitoneum deflation and the smoke plume that can be produced during laparoscopic electrocautery and the use of high-energy devices [9]. The Colleges also recommended the use of non-operative management (NOM) in certain cases and the preference for open appendicectomies when operative management was required [9].

The aim of our study was to compare the outcomes of treating acute appendicitis surgically (laparoscopic versus open) in terms of clinical improvement, hospital stay, sepsis and pain control during COVID-19 pandemic.

## Materials and methods

Study design

The Dudley Group NHS Foundation Trust served as the site for this retrospective cohort study. The records were kept in the form of admission notes, operation notes, drug charts, sunrise system and discharge notes. The study was approved by the medical ethics committee at The Dudley Group NHS Foundation Trust, which also decided that informed consent was not required. Patient identity was kept anonymous. Excel (Microsoft, Redmond, WA, USA) was used to collect retrospectively and analyse data during November 2019 to July 2021.

These patient files were manually screened and chosen following the initial selection. The following symptoms, signs, and investigation findings had to be met in order for the patient to be considered for inclusion: severe right iliac fossa pain and tenderness, abdominal rigidity and rebound tenderness along with Rovsing's sign positive.

Pregnant women, non-consenting individuals, patients with severe sepsis or septic shock upon presentation, patients with an appendix mass or abscess unsuitable for laparoscopic appendectomy, and patients with intellectual disabilities that impede the acquisition of informed consent were among the exclusion criteria. Surgical conversion patients were not included. Additionally, patients with a postoperative pathological diagnosis of carcinoma, midline laparotomy, and extended resection of the colon or caecum (ileocecal resection or right hemicolectomy) were not included.

Data for laparoscopic and open appendicectomy such as gender, age, duration of right iliac fossa pain, intraoperative findings (perforation, inflammation and other findings), histology, complications, type and duration of antibiotics, length of stay and readmission were anlaysed.

Informed verbal and written consent was procured from the patients in both groups. Participants received in-depth counselling about the drawbacks, dangers, and benefits of each intervention.

Using the Hasson method to create pneumoperitoneum, a standard three-port laparoscopic appendectomy was carried out. The mesoappendix was dissected using electrocautery. Using laparoscopic scissors, the appendicular base was knotted and divided between two Ethicon endo-loops. With the use of an extraction bag, the dissected tissue was removed. The resultant stump of the appendix was not buried in a regular manner. The Gridiron incision was used in a standard manner for the open appendectomy procedure. The mesoappendix was the site of the ligation. The tissue was then removed after dividing the appendicular base. There was no burial for the appendicular stump. Every tissue sample that was collected was examined under a microscope.

Data collection

Data for patients with acute appendicitis were gathered retrospectively from operation reports, admission charts, and discharge reports which were utilized to gather the necessary data, which was then processed anonymously and analyzed.

## Results

The total number of patients enrolled in this study was 80, with an equal number of patients having undergone open and laparoscopic appendectomy (40 each). Twenty-six (65% of open) females and 14 (35% of open) males underwent open appendectomy, while the gender ratio of those who underwent laparoscopic appendectomy was almost equivalent (21 (52.5%) and 19 (47.5%) respectively). The mean age of patients undergoing laparoscopic appendectomy was higher at 34 years compared to the average of 32 years in open appendectomy and the overall postoperative complication rate was higher in the laparoscopic group (22%, compared to 18% in the open group) as illustrated in Table 1.

**Table 1:** Demographic data

Type of Appendicectomy	Number of Cases (%)	Gender (%)	Mean/Median Age in Years	Readmission (%)	Post- operative Complications
Open	40 (50%)	Male - 14 (35%)	32/26	4 (10%)	7 (17.5%)
		Female – 26 (65%)			
Laparoscopic	40 (50%)	Male – 21 (52.5%)	34/40	3 (7.5%)	9 (22.5%)
		Female – 19 (47.5%)			

Table 2 shows that the duration of symptoms for right inferior quadrant pain is more prolonged in the laparoscopic group.

**Table 2:** Duration of Right Inferior Quadrant Pain

Number of Days	Open n - (%)	Laparoscopic - n (%)	Total n (%)
1	9 (22%)	14 (35%)	23 (29%)
2	7 (17%)	5 (12%)	12 (15%)
3	1 (3%)	4 (10%)	5 (6%)
4+	1 (3%)	2 (5%)	3 (4%)
Not Recorded	22 (55%)	15 (38%)	37 (46%)
Total	40 (100%)	40 (100%)	80 (100%)

The laparoscopic group had more severe Intraoperative findings (20% normal in laparoscopic, and 35% in open appendectomy) as illustrated in Table 3.

**Table 3:** Intraoperative FIndings The Laparoscopic Group had more severe intraoperative findings

Type Of Appendicectomy	Normal	Inflamed	Perforated	Other	p-Value
Open - n (%)	14 (35%)	19 (47.5%)	3 (7.5%)	4 (10%)	
Laparoscopic -n (%)	8 (20%)	25 (62.5%)	4 (10%)	3 (7.5%)	
Total – n (%)	22 (27.5%)	44 (55%)	7 (8.8%)	7 (8.8%)	0.03

Upon histology acute suppurative appendicitis, which is indicative of severe and prolonged appendicitis, was more commonly found in open appendectomy (65%, with only 42.5% such cases in laparoscopy) as demonstrated in Table 4. The rate of open wound infection was more in the open appendectomy group as demonstrated in Table 5.

**Table 4:** Histological Findings Upon histology, acute suppurative appendicitis, which is indicative of severe and prolonged appendicitis, was more commonly found in open appendectomy (65%, with only 42.5% such cases in laparoscopy).

Type Of Appendicectomy	Acute Appendicitis	Acute Suppurative Appendicitis	Acute Ulcerative Appendicitis	Hyperplasia	Transmural Inflammation	Mucosal Ulceration	Severe Appendicitis	No significant histology	Not Recorded	p-Value
Open – n (%)	5 (12.5%)	26 (65%)	0 (0%)	6 (15%)	2 (5%)	0 (0%)	0 (0%)	0 (0%)	1 (2.5%)	
Laparoscopic – n (%)	8 (20%)	17 (42.5%)	1 (2.5%)	3 (7.5%)	3 (7.5%)	3 (7.5%)	1 (2.5%)	2 (5%)	2 (5%)	
Total – n (0%)	13 (16.25%)	43 (53.75%)	1 (1.25%)	9 (11.25%)	5 (6.25%)	3 (3.75%)	1 (1.25%)	2 (2.5%)	3 (3.75%)	0.07

**Table 5:** Breakdown of Post-operative Complications

Type Of Appendicectomy	Acute Kidney Injury (%)	Ileus (%)	Wound Infection (%)	Vessel Bleed (%)	Antibiotic Allergy (%)	Collection (%)	Hypokalaemia (%)	Post operative drain (%)	High Temperature (%)	p-Value
Open	6 (15%)	11 (27.5%)	17 (42.5)	6 (15%)	0 (0%)	0 (0%)	0 (0%)	0 (0%)	0 (0%)	
Laparoscopic		9 (22.5%)	0 (0%)	0 (0%)	5 (12.5%)	9 (22.5%)	5 (12.5%)	9 (22.5%)	3 (7.5%)	
Total	6 (7.5%)	20 (25%)	17 (21.25%)	6 (7.5%)	5 (6.25%)	9 (11.25%)	5 (6.25%)	9 (11.25%)	3 (3.75%)	0.35

More patients were discharged with antibiotics in the open group, and this group also had a prolonged duration of antibiotic usage, the most common in both being co-amoxiclav as illustrated in Table 6. Table 7 shows the duration of antibiotics prescribed post-operation.

**Table 6:** Antibiotics prescribed post-operation

Type Of Appendicectomy	Co-amoxiclav	Co-amoxiclav and Metronidazole	Co-trimaxole and Metronidazile	Doxycycline and Penicillin	Ciprofloxacillin	Ciprofloxacillin and Metronidazole	Flucloxacillin and Metronidazole	Amoxacillin and Metronidazole	Not Recorded
Open	29 (72.5%)	0 (0%)	0 (0%)	0 (0%)	5 (12.5%)	4 (10%)	4 (10%)	4 (10%)	4 (10%)
Laparoscopic	32 (80%)	2 (5%)	2 (5%)	2 (5%)	0 (0%)	0 (0%)	0 (0%)	0 (0%)	2 (5%)

**Table 7:** Duration of antibiotics post operation

Type Of Appendicectomy	1-3 days (%)	4-5 days (%)	6-7 days (%)	8+ days (%)	Not recorded (%)
Open	7 (17.5%)	24 (60%)	5 (12.5%)	0 (0%)	4 (10%)
Laparoscopic	4 (10%)	27 (67.5%)	7 (17.5%)	2 (5%)	0 (0%)

## Discussion

Quah et al. conducted a recent analysis of three randomized-control trials, which revealed that although laparoscopic appendectomy shows a statistically significant decrease in death and disability, open appendectomy is currently the more common procedure performed for complicated appendicitis due to a reported higher incidence of intra-abdominal abscess (IAA) formation [10,11]. When compared to open appendectomy, laparoscopic appendectomy is also linked to better health outcomes and a shorter duration of hospital stay [10,11]. Additionally, they discovered comparable IAA statistics for the two groups [10,11]. Subsequently, the researchers suggested laparoscopic appendectomy in cases of severe appendicitis [10,11]. This is at odds with our results, which indicated that there was no statistically significant difference in postoperative health outcomes between laparoscopic and open appendectomy [10,11].

A retrospective study by Horvath et al. compared laparoscopic and open appendicectomies for perforated appendicitis, involving 1,762 patients [11,12]. They discovered that although surgical site infections (SSIs) were a postoperative complication that only happened to patients who had open appendices, the incidence of IAA was statistically significant (p-value of 0.002) in patients who had laparoscopic appendices [11,12]. Additionally, they mentioned a shorter hospital stay following a laparoscopic appendectomy [11,12]. Surgeons should remember the actions that can be taken, like irrigations, stump handling, and endo bag use, to lessen the formation of IAA, according to this study's recommendations [11,12].

Studies by Yu et al., Wullsteain et al. and Ball et al. all found laparoscopic appendectomy better in terms of patients outcome especially in complicated cases [13,14]. The incidence of complicated appendicitis did not change during the COVID-19 pandemic. Although the number of patients with acute appendicitis decreased, prominent changes in the rate of complicated appendicitis were not observed, indicating that patients sought ED help when they had abdominal pain [15].

According to research, there were no appreciable changes in the majority of complications, including intra-abdominal collections, re-admission, re-operations, and bleeding, between the two groups during COVID-19 [16]. Thus, provided appropriate safety measures are followed, both laparoscopic and open appendectomies seem to be practical and safe procedures. It was intriguing to see that the study's SSI rate was much greater for appendectomies done during the epidemic [16].

Another study reported that there was no increase in the incidence of severe appendicitis, surgical complications, or length of stay, even though patients presented later and there was a minor delay between admission and surgical therapy during the pandemic [17]. An excellent prognosis for appendicitis was made possible by prompt clinical diagnosis, resuscitation, antibiotics, and surgical care [17]. It was also demonstrated that a safe and efficient standard for the treatment of appendicitis during the epidemic was laparoscopic appendicectomy.

The debate over whether laparotomy or laparoscopy was preferable persisted, given the danger of COVID-19 spreading [18]. Because laparoscopic surgery produced a larger concentration of surgical smoke than open surgery, there was a documented increase in the risk of infection among surgical workers [18]. However, if sufficient infection control measures were implemented, laparoscopic surgery also indicated a decreased risk of infection [18].

Based on the available literature and the above, our research indicates that laparoscopic appendectomy could potentially yield better and safer health outcomes, provided that appropriate technique, patient risk factor stratification, and postoperative care are ensured. However, the limitations of this study prevent a more thorough analysis, and more research is necessary to influence policy.

Limitations

This was a retrospective and an audit study. This highlights some key points, which can be looked into through further clinical trials for further evidence.

## Conclusions

In conclusion, our study offers valuable insights into the outcomes of acute appendicitis, emphasizing the need for a nuanced understanding of patient demographics and clinical presentations when comparing open and laparoscopic appendectomy. The disparities in complication rates and postoperative management underscore the complexity of decision-making in surgical approaches. Further research is warranted to explore the underlying factors contributing to these differences and to refine guidelines for optimal patient care, especially in the context of challenging healthcare scenarios such as the COVID-19 pandemic.
